# Neutrophil-to-lymphocyte ratio as a predictor of in-hospital complications and overall mortality in Takotsubo syndrome preceded by physical triggers

**DOI:** 10.1186/s12872-023-03078-1

**Published:** 2023-01-27

**Authors:** Hyo-Jeong Ahn, Jeehoon Kang, So-Ryoung Lee, Jin Joo Park, Hae-Young Lee, Dong-Ju Choi, Hyun-Jai Cho

**Affiliations:** 1 Division of Cardiology, Department of Internal Medicine, Seoul National University Hospital, 101 Daehak-ro, Jongno-gu, Seoul, 03080 Republic of Korea; 2 Department of Critical Care Medicine, Seoul National University Hospital, Seoul, Republic of Korea; 3 Department of Internal Medicine, Seoul National University Bundang Hospital, Seongnam, Republic of Korea

**Keywords:** Takotsubo syndrome, Neutrophil-to-lymphocyte ratio, In-hospital complication, Mortality

## Abstract

**Background:**

Takotsubo syndrome (TTS) with physical triggers has worse short- and long-term clinical courses than those with emotional triggers. However, predictive factors associated with poor outcomes of TTS with physical triggers are unknown.

**Methods:**

We included 231 patients identified as TTS preceded by physical triggers at two tertiary referral hospitals from 2010 to 2019. In-hospital complications (IHC)—a composite of malignant arrhythmia, need for mechanical circulatory support or mechanical ventilation, and in-hospital death—and overall mortality were retrospectively reviewed. The associations with clinical features were evaluated by multivariable logistic and Cox regression analyses.

**Results:**

The mean age was 69.3 ± 11.6 years, and 85 (36.8%) were male. The in-hospital complications rate was 46.8%. During a median follow-up of 883 days, 96 (41.6%) had died, and overall mortality was 13.6% per patient-year. Higher neutrophil-to-lymphocyte ratio (NLR) was associated with a higher risk of IHC (area under the receiver operating characteristic curve = 0.73; positive and negative predictive value = 60.9% and 67.2% for NLR ≤ 12); odds ratio (OR) with 95% confidence interval (CI) was 1.03 (1.01–1.05), p = 0.010. Subsequently, higher NLR was also related to a greater risk of overall mortality; patients with high NLR (NLR > 12) exhibited poor long-term survival than those with low NLR (NLR ≤ 5): hazard ratio (95% CI), 3.70 (1.72–7.94) with p < 0.001.

**Conclusions:**

A high NLR at initial presentation is associated with an increased risk of IHC and overall mortality in TTS preceded by physical triggers. Given that the treatment of TTS is mainly supportive, intensive monitoring with careful follow-up would be warranted in patients with high NLR.

**Supplementary Information:**

The online version contains supplementary material available at 10.1186/s12872-023-03078-1.

## Introduction

Takotsubo syndrome (TTS) is an acute, reversible form of heart failure characterized by transient systolic and diastolic left ventricular dysfunction with various regional wall-motion abnormalities [1, 2]. Despite various explanations of the underlying mechanisms of TTS, such as myocardium stunning by stress-related neuropeptides, sympathetic overstimulation, microvascular spasm, and endothelial dysfunction, the exact pathophysiology of the condition remains unclarified [3, 4]. In approximately two-thirds of cases, TTS is preceded by an emotional or physically stressful event [5] and physical triggers are reported to be more frequent than emotional triggers [2, 6].

Historically, patients with TTS were expected to have favorable outcomes; however, recent studies have challenged this by reporting a substantial risk of in-hospital complications (IHCs) and long-term mortality [7, 8]. About 25% of patients with TTS experience complications during hospitalization, mainly related to cardiovascular adverse events [1, 2]. Moreover, up to 5% of in-hospital mortality and higher delayed mortality after hospital discharge than a general population were reported [3, 7, 9]. Especially, TTS with physical stressors was revealed to have more adverse outcomes of both in-hospital course and long-term mortality compared to those with emotional triggers [6, 10].

Several presenting features such as older age, male sex, acute neurologic disorders, atypical ballooning, high troponin levels, and low left ventricular ejection fraction (LVEF) are associated with poor short and long-term prognoses in TTS [1, 2, 6]. However, there is a paucity of information on whether these characteristics sustain the predictive value for adverse clinical outcomes in TTS preceded by physical triggers which was shown to have distinct outcomes from that of emotional triggers. Therefore, we investigate clinical characteristics predicting IHCs and in turn, evaluate the associations with long-term mortality in TTS accompanied by physical triggers.

## Methods

### Study population

This is a multicenter retrospective cohort study including hospitalized patients who were diagnosed with TTS at two tertiary hospitals (Seoul National University Hospital and Seoul National University Bundang Hospital, Korea) from January 1st, 2010 to December 31st, 2019. All patients initially met the diagnostic criteria of TTS clinically and echocardiographically, and also underwent coronary artery evaluation (i.e., invasive coronary angiography, coronary computed tomographic angiography, and myocardial perfusion scanning) [11]. Then, we included patients who fulfilled the revised diagnostic criteria of Mayo Clinic for this condition, as follows: a transient abnormality of wall motion in the left ventricle beyond a single epicardial coronary artery perfusion territory, the absence of culprit obstructive coronary artery disease or angiographic evidence of acute plaque rupture which could explain the wall motion abnormality, new electrocardiographic abnormalities or elevation in cardiac troponin levels, and the absence of pheochromocytoma and myocarditis [12, 13]. For the preceding factor, we defined physical triggers as any acute medical illness that could induce alterations in individuals’ overall well-being (i.e., acute respiratory failure, infection, post-surgical problems, malignancy, or stroke) and emotional triggers as any recent psychological stressful events in the absence of evident physical triggers (i.e., death of a family member, tragic news, or trouble in personal relationships). We only included those without a definite emotional trigger. Any case of disagreement in the diagnosis of TTS was thoroughly reviewed by two independent investigators to reach a consensus. Institutional Review Board at the Seoul National University Hospital (E-2003-071-1108) authorized this study and written informed consent was waived in all subjects.

### Baseline characteristics

Demographic factors, comorbidities, and physical stress assumed to trigger TTS were reviewed from medical records. Initial clinical presentation, vital signs, laboratory values checked during admission, and inflammatory markers such as white blood cell (WBC) count were also investigated. The neutrophil-to-lymphocyte ratio (NLR) was calculated by dividing the absolute number of each WBC subtype and reported from complete blood and its differential count. The patients underwent several CBC measurements at short intervals from the initial diagnosis of TTS due to their acute illness and alteration in its profile. Typically, it was monitored every 24 h, and more frequently for those with anemia or other abnormal cell counts. Therefore, we defined the neutrophil-to-lymphocyte ratio (NLR) as a peak value reported within 48 h of the first recognition of TTS to reflect the disease status of individuals [14]. The platelet-to-lymphocyte ratio (PLR) was also investigated in the same manner as another hematologic index. Electrocardiogram, LVEF with a type of regional wall motion abnormalities from the 2-dimensional echocardiography, and the results of coronary artery evaluation were reviewed as well.

### Definition of outcome

The primary outcomes of our study were the occurrence of IHCs and long-term mortality. We defined IHCs as a composite of malignant arrhythmia (atrial fibrillation with rapid ventricular response accompanying low blood pressure, ventricular tachycardia, ventricular fibrillation, torsade de pointes, asystole, and complete atrioventricular block), a need for mechanical circulatory support (i.e., intra-aortic balloon pump or extracorporeal membrane oxygenation) or mechanical ventilation, and in-hospital death in reference to previous publications exploring clinical courses of TTS [1, 15]. The overall mortality record was retrieved from the national statistical information service and last queried on August 25th, 2020. Data analysis was performed from September 2020 to November 2020. Clinical characteristics associated with IHCs and overall mortality were investigated by multivariable logistic regression and Cox proportional hazards regression models, respectively. Subsequently, the variable both commonly associated with IHCs and overall mortality was found and its predictive value was evaluated by the analysis of the receiver operating characteristic curve.

### Statistical analysis

Continuous variables were reported as means ± standard deviations or medians (interquartile ranges) and categorical variables as number (%). Normal distribution of variables was identified by Kolmogorov–Smirnov test. Student’s *t* test or Mann–Whitney test was performed to compare continuous variables, and Pearson chi-square test or Fisher’s exact test was applied for categorical variables as required. Variables shown to be associated with IHCs in the univariable testing (p < 0.05) and previously reported prognostic factors were subsequently evaluated in multivariable logistic regression; age, sex, cancer expected lifespan < 6 months, chest pain, peak troponin I, NLR, peak C-reactive protein (CRP), LVEF, and atypical ballooning. The IHC risk of each clinical factor was presented as odds ratio (ORs) with 95% confidence intervals (CIs). The relationship with overall mortality was assessed by Cox proportional hazards regression models adjusting the same variables in the multivariable logistic regression and presented as hazard ratios (HR) with 95% CIs. To examine survival estimates, Kaplan–Meier method was used and the log-rank test or the Breslow test was assessed to compare groups. Sensitivity analyses were performed to evaluate the association among clinical features, the risk of in-hospital complications, and overall mortality by excluding those with underlying cancer of expected lifespan < 6 months. We also performed several subgroup analyses of overall mortality according to age (< 70 and ≥ 70 years, divided by the median value), sex, and baseline LVEF (≤ 40 and > 40%) to verify our result's robustness. The p-for-interaction was estimated to confirm whether the main result remains consistent across subgroups. P < 0.05 (two-tailed) was considered statistically significant. All analyses were performed using SPSS statistical software, version 26.0 and Stata (version 14.2, StataCorp LLC, College Station, TX, USA).

## Results

### Baseline characteristics

**Fig. 1:**
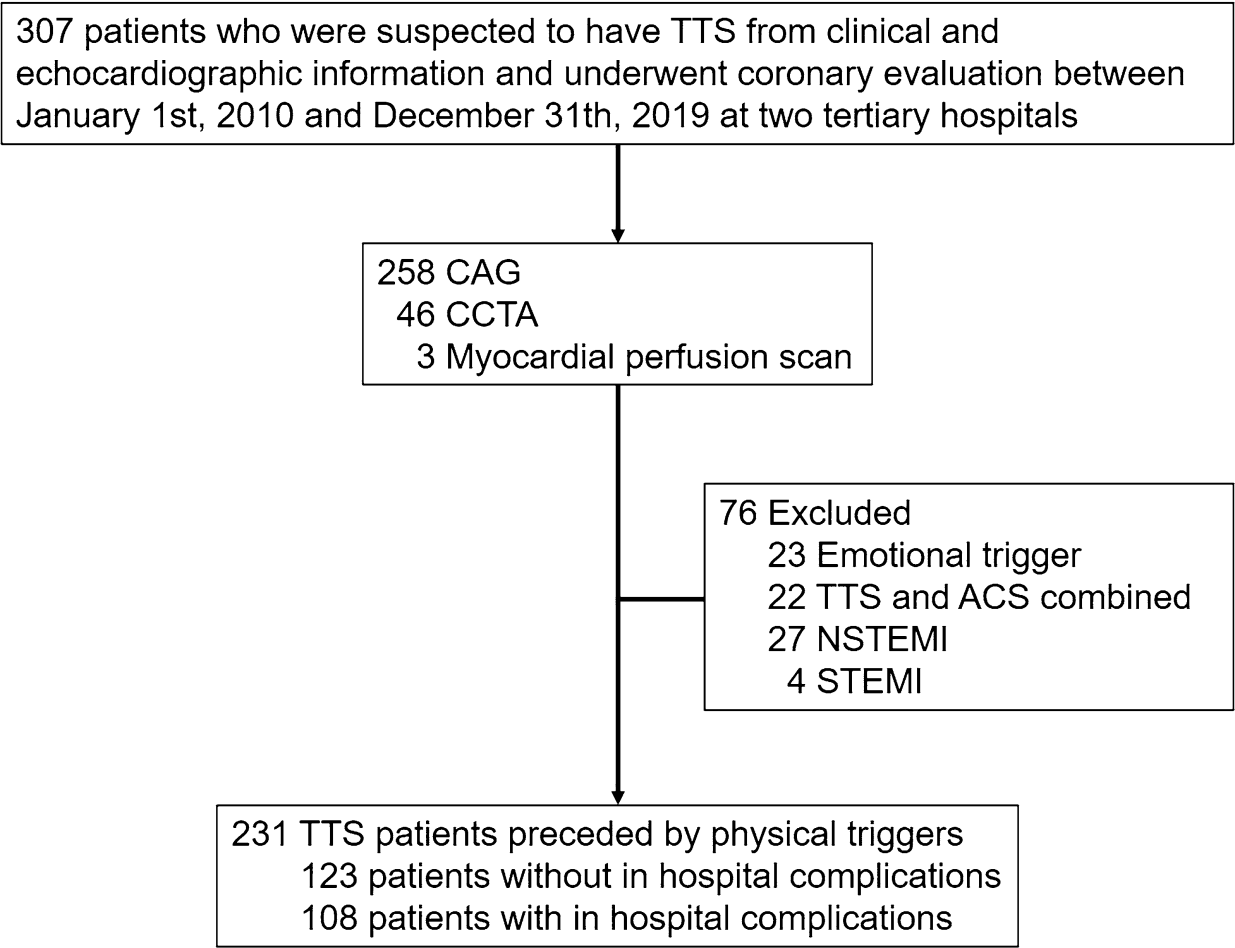
Selection of patients with Takotsubo syndrome preceded by physical triggers. TTS, Takotsubo syndrome; CAG, coronary angiography; CCTA, coronary computed tomography angiography; ACS, acute coronary syndrome; NSTEMI, Non-ST segment elevation myocardial infarction; STEMI, ST segment elevation myocardial infarction

A total of 231 patients with TTS preceded by physical triggers were included in the analysis (Fig. 1). Baseline features of our study population according to the presence of IHCs are summarized in Table 1 and detailed information is presented in Additional file 1: Table S1. Also, the general clinical aspects of 23 excluded TTS patients preceded by emotional triggers are summarized in Additional file 1: Table S2. Compared to TTS patients with emotional triggers, those with physical triggers presented a higher baseline NLR value (median 12.0 vs. 3.9, p = 0.008), a lower baseline LVEF (39.7% vs. 45.7%, p = 0.016), and a higher intensive care unit (ICU) admission rate (51.9% vs. 27.8%, p = 0.048) (Additional file 1: Table S2).

**Table 1:** Baseline characteristics of the study population according to the presence of in-hospital complications

	Total (n = 231)	No In-hospital complication (n = 123)	In-hospital complication (n = 108)	P value
Age	69.31 ± 11.61	69.74 ± 12.28	68.81 ± 10.83	0.547
Male	85 (36.8)	33 (26.8)	52 (48.1)	0.001
Body mass index (kg/m^2^)	21.61 ± 3.86	21.68 ± 3.84	21.53 ± 3.90	0.770
Comorbidities				
Hypertension	100 (43.3)	58 (47.2)	42 (38.9)	0.206
Diabetes mellitus	64 (27.7)	37 (30.1)	27 (25.0)	0.389
Dyslipidemia	19 (8.2)	11 (8.9)	8 (7.4)	0.672
Coronary artery disease	40 (17.3)	23 (18.7)	17 (15.7)	0.553
COPD or asthma	22 (9.5)	12 (9.8)	10 (9.3)	0.898
ESRD	19 (8.2)	10 (8.1)	9 (8.3)	0.955
Chronic liver disease	10 (4.3)	3 (2.4)	7 (6.5)	0.195
Cancer expected lifespan < 6 months	39 (16.9)	15 (12.2)	24 (22.2)	0.042
Neurologic disorders	68 (29.4)	37 (30.1)	31 (28.7)	0.819
Trigger				
After procedure or operation	31 (13.4)	17 (13.8)	14 (13.0)	0.248
Neurologic problems	35 (15.2)	23 (18.7)	12 (11.1)	
Others	165 (71.4)	83 (67.5)	82 (75.9)	
Clinical presentation and vital sign				
Chest pain	62 (26.8)	43 (35.0)	19 (17.6)	0.003
Dyspnea	84 (36.4)	31 (25.2)	53 (49.1)	< 0.001
Initial vital signs				
SBP (mmHg)	110.35 ± 31.06	117.44 ± 30.74	102.27 ± 29.54	< 0.001
DBP (mmHg)	67.81 ± 17.46	71.27 ± 17.43	63.87 ± 16.71	0.001
Heart rate (/min)	98.11 ± 24.56	92.53 ± 22.06	104.47 ± 25.78	< 0.001
Laboratory values				
Initial Troponin I (ng/mL)	0.43 (0.07–2.21)	0.40 (0.06–2.16)	0.47 (0.10–2.28)	0.324
Peak Troponin I (ng/mL)	2.01 (0.43–5.43)	1.29 (0.28–3.40)	2.93 (0.96–8.46)	< 0.001
Initial CRP (mg/dL)	4.30 (0.69–14.10)	3.12 (0.52–7.03)	6.68 (1.59–19.37)	0.001
Peak CRP (mg/dL)	10.47 (3.88–19.87)	5.88 (1.27–13.46)	17.87 (8.04–25.99)	< 0.001
NLR	11.95 (5.40–22.50)	6.74 (3.98–16.31)	16.42 (9.57–32.08)	< 0.001
PLR	178.75 (98.22–303.29)	164.54 (91.19–299.44)	186.49 (105.91–322.12)	0.096
ECG				
Sinus rhythm	195 (84.4)	101 (82.1)	94 (87.0)	0.303
Atrial fibrillation	21 (9.1)	13 (10.6)	8 (7.4)	0.404
Echocardiographic finding				
LVEF (%)	39.70 ± 10.96	42.03 ± 9.87	37.04 ± 11.57	0.001
Apical ballooning	169 (73.2)	89 (72.4)	80 (74.1)	0.769

COPD, chronic obstructive pulmonary disease; ESRD, end stage renal disease; SBP, systolic blood pressure; DBP, diastolic blood pressure; CKMB, Creatine kinase-MB; CRP, C-reactive protein; WBC, white blood cell; NLR, neutrophil-to-lymphocyte ratio; LVEF, left ventricular ejection fraction

**Table 2:** Predictors of in-hospital complications

Variables	Univariable analysis		Multivariable analysis	
	Odds ratio (95% CI)	P value	β (95% CI)	P value
Age*	0.93 (0.75–1.17)	0.545	0.93 (0.70–1.22)	0.583
Male sex	2.53 (1.46–4.39)	0.001	1.58 (0.82–3.04)	0.169
Cancer expected lifespan < 6 months	2.06 (1.02–4.17)	0.045	1.17 (0.50–2.75)	0.722
Chest pain	0.40 (0.21–0.74)	0.003	0.48 (0.23–1.00)	0.050
Peak troponin I	1.03 (1.00–1.07)	0.033	1.03 (0.99–1.06)	0.121
NLR	1.04 (1.02–1.06)	< 0.001	1.03 (1.01–1.05)	0.010
Peak CRP	1.09 (1.06–1.12)	< 0.001	1.07 (1.03–1.10)	< 0.001
LVEF	0.96 (0.93–0.98)	0.001	0.96 (0.94–0.99)	0.019
Atypical ballooning	0.92 (0.51–1.64)	0.769	0.86 (0.41–1.80)	0.686

CI, confidence interval; NLR, neutrophil-to-lymphocyte ratio; CRP, C-reactive protein; LVEF, left ventricular ejection fraction

*For a 10-year increase

Furthermore, out of the 231 patients with TTS preceded by physical triggers, the mean age was 69.3 ± 11.6 years and 146 (63.2%) were women. Overall IHCs rate was 46.8% (108 of 231): malignant arrhythmia, 27 (11.7%), mechanical circulatory support 13 (5.6%), mechanical ventilation 92 (39.8%), and in-hospital death 27 (11.7%). The causes of in-hospital death were primarily due to non-cardiac (85.2%); 4 cases (14.8%) were cardiac deaths, 11 cases (40.7%) were due to respiratory failure, 4 cases (14.8%) were due to sepsis, and 8 cases (29.6%) were death owing to the progression of underlying disease (e.g., cancer progression), respectively. Triggers of TTS were categorized into three groups: procedure or operation related (e.g., bowel surgery, intrathoracic surgery, vascular surgery, or organ transplantation, n = 31, 13.4%), neurologic problems related (e.g., intracranial bleeding, ischemic stroke, seizures, or encephalitis, n = 35, 15.2%), and the others. A more detailed classification of triggers is described in Additional file 1: Fig. S1. Among physical triggers other than procedure or operation-related and neurologic problems, acute respiratory failure (n = 74, 32.0%) and infectious problems (intra-abdominal infection, n = 29, 12.6% and other infection, n = 7, 3.5%) were the most frequent acute illness followed by the development of TTS. The predominant symptoms at the first recognition of the disorder were dyspnea (36.4%) and chest pain (26.8%), whereas other nonspecific symptoms or incidental findings such as dizziness, delirious behavior, sustained tachycardia, or T wave inversion during monitoring accounted for approximately one-third of cases. The median NLR value of patients with IHCs was 16.4 (9.6–32.1) which was significantly higher than the value of patients without IHCs, 6.7 (4.0–16.3). Almost half of the patients showed reduced LVEF (≤ 40%) at initial presentation of which the majority were those with IHCs. Follow-up echocardiographic evaluation was available for 167 (72.3%) patients with a median interval of 20.5 (8.4–85.6) days (Additional file 1: Table S3). In accordance with the definition of TTS, recovery of partial or complete regional wall motion abnormality was observed in 149 (89.2%) patients. An improvement in LVEF was identified in 155 (92.8%) patients with no difference in the degree of increment between in-hospital survivors and the others (15.3% increase in LVEF, p = 0.99).

**Table 3:** Predictors of overall mortality

Variables	Univariable analysis		Multivariable analysis	
	Hazard ratio (95% CI)	P value	Hazard ratio (95% CI)	P value
Age*	1.23 (1.02–1.48)	0.033	1.29 (1.05–1.57)	0.014
Male sex	1.58 (1.06–2.36)	0.026	1.33 (0.87–2.03)	0.195
Cancer expected lifespan < 6 months	2.21 (1.39–3.53)	0.001	1.51 (0.90–2.54)	0.122
Chest pain	1.01 (0.64–1.58)	0.979	1.41 (0.87–2.29)	0.164
Peak troponin I	1.00 (1.00–1.01)	0.402	1.00 (1.00–1.01)	0.200
NLR	1.02 (1.01–1.03)	< 0.001	1.01 (1.00–1.02)	0.006
Peak CRP	1.03 (1.01–1.05)	0.001	1.01 (0.99–1.04)	0.214
LVEF	0.97 (0.95–0.99)	0.002	0.98 (0.96–1.00)	0.056
Atypical ballooning	1.01 (0.65–1.58)	0.956	1.09 (0.69–1.71)	0.723

CI, confidential interval; NLR, neutrophil-to-lymphocyte ratio; CRP, C-reactive protein; LVEF, left ventricular ejection fraction

*For a 10-year increase

### Predictors of in-hospital complications and long-term mortality

In univariable analysis, sex, underlying cancer expected lifespan < 6 months, initial clinical presentations and vital signs, peak cardiac enzymes, inflammatory markers, presence of ST-segment elevation, and LVEF were associated with IHCs. We included representative factors of each clinical category along with known prognostic factors (i.e., age and atypical ballooning) in multivariable analysis. Finally, independent factors identified as to be significantly correlated with IHCs are summarized in Table 2: NLR (OR, 1.03; 95% CI, 1.01–1.05; p = 0.010), peak CRP (OR, 1.07; 95% CI, 1.03–1.10; p < 0.001), and LVEF (OR, 0.96; 95% CI, 0.94–0.99; p = 0.019).

Variables evaluated in the multivariable logistic regression model were subsequently computed in Cox proportional hazards regression models and NLR was demonstrated to be a predictor of overall mortality, as well (Table 3): adjusted HR (95% CI), 1.29 (1.05–1.57) for an age of 10-year increase (p = 0.014) and 1.01 (1.00–1.02) for NLR (p = 0.006).

### Neutrophil-to-lymphocyte ratio as a predictor of clinical courses of TTS

**Fig. 2:**
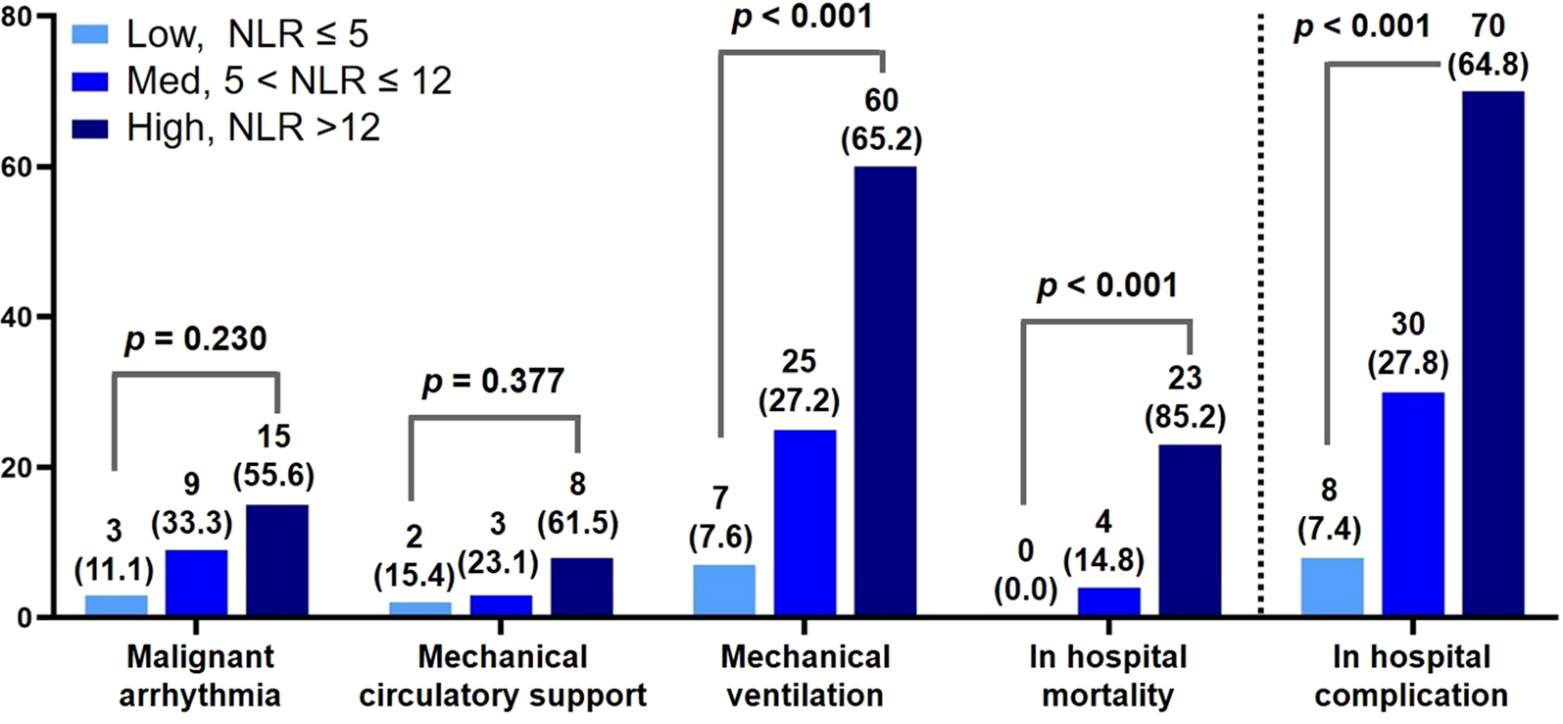
The number of patients with individual components of in hospital complications stratified according to the value of neutrophil-to-lymphocyte ratio (NLR) values (Low, NLR ≤ 5; Med, 5 < NLR ≤ 12; High, NLR > 12). NLR, neutrophil-to-lymphocyte ratio

The receiver operating characteristic curve of NLR regarding IHCs is described in Additional file 1: Fig. S2. The area under the curve is 0.73 (95% CI 0.67–0.79, p < 0.001). The negative and positive predictive values are 67.2% and 60.9%, respectively with an NLR value of less than 12, which was determined by the highest sensitivity and specificity to classify whether a patient has IHC or not (sensitivity, 64.8%; specificity, 63.4%). Also, as NLR > 5 was considered elevated in the general population according to the published literature [16, 17], we divided the study population into three groups with discrimination values of NLR 5 and 12; the number of patients with low NLR (NLR ≤ 5) was 53, medium NLR (5 < NLR ≤ 12) was 63, and high NLR (NLR > 12) was 115. The numbers of patients with individual components of IHCs stratified according to the value of NLR 5 and 12 were evaluated and presented in Fig. 2. Increased measurement of NLR with discrimination values 5 and 12 is associated with a higher risk of mechanical ventilation, in-hospital mortality, and IHC (p < 0.001, respectively). Additional file 1: Fig. S3 demonstrated the number of patients with the presence of individual components of IHCs stratified according to the value of NLR as 12. Among IHCs, the value of NLR 12 was associated with the need for mechanical ventilation (OR, 2.86; 95% CI 1.66–4.95; p < 0.001) and in-hospital mortality (OR, 7.00; 95% CI 2.34–20.97; p < 0.001).

### Survival analysis

**Fig. 3:**
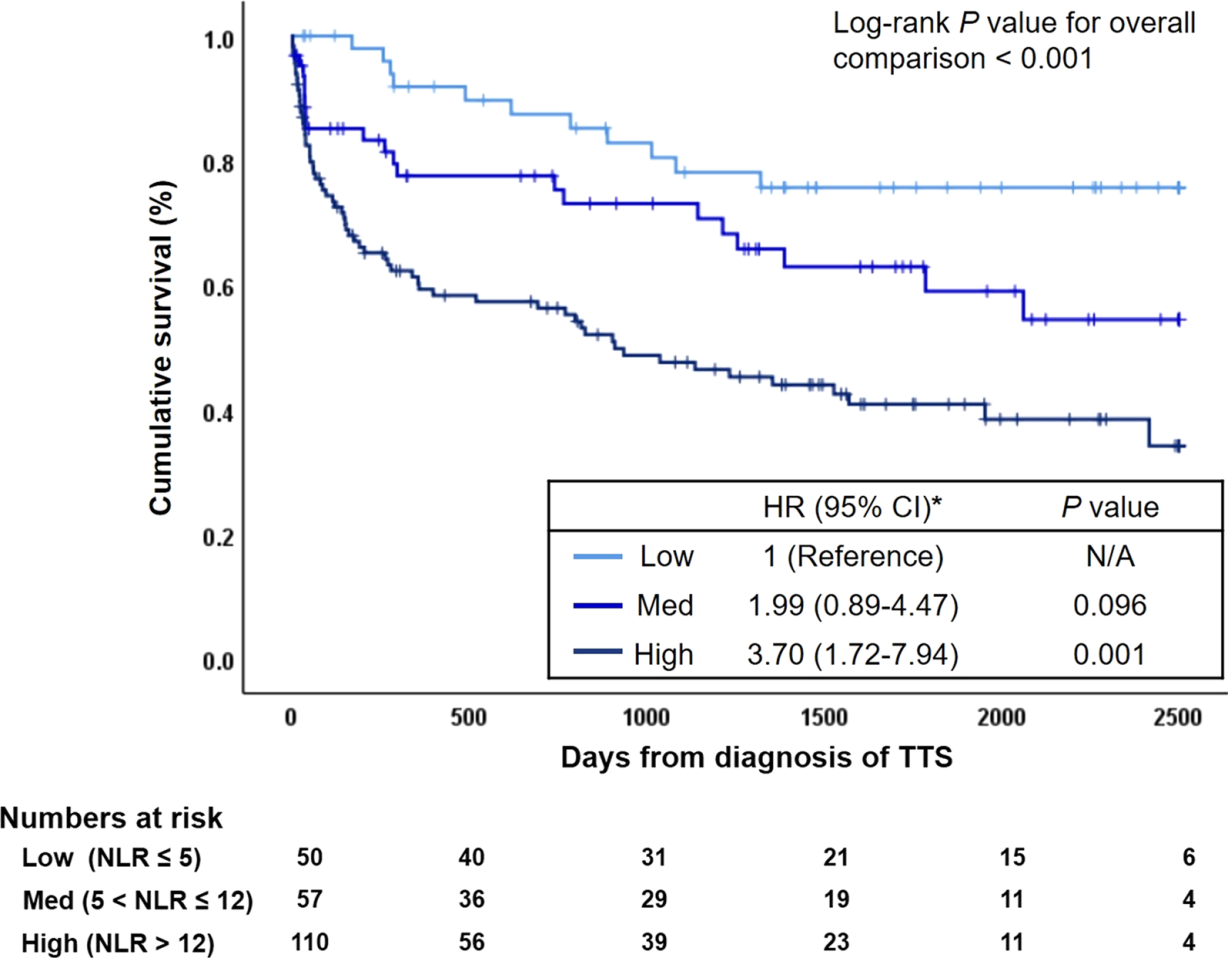
Kaplan–Meier survival curves of the patients of Takotsubo syndrome with physical triggers separated on the basis of neutrophil-to-lymphocyte ratio (NLR) values (Low, NLR ≤ 5; Med, 5 < NLR ≤ 12; High, NLR > 12). *Adjusted for age, sex, cancer expected lifespan < 6 months, chest pain, peak TnI, peak CRP, LVEF, and atypical ballooning. TTS, takotsubo syndrome; NLR, neutrophil-to-lymphocyte ratio; HR, hazard ratio; CI, confidence interval; TnI, troponin I; CRP, C-reactive protein; LVEF, left ventricular ejection fraction

Patients with TTS preceded by physical triggers were followed up during a median of 883 days (interquartile range, 143–1779) and 96 (41.6%) had died. The overall mortality rate was 13.6% per patient-year. The Kaplan–Meier analysis of long-term survival is illustrated in Fig. 3 and Additional file 1: Fig. S4. The long-term survival of patients with high NLR value (NLR > 12) and medium NLR value (5 < NLR ≤ 12) at initial presentation was significantly worse than the patients with low NLR value (NLR ≤ 5): adjusted HR (95% CI), 3.70 (1.72–7.94) for high NLR and 1.99 (0.89–4.47) for medium NLR value with each p-value 0.001 and 0.096, respectively.

### Sensitivity and subgroup analyses

Sensitivity analyses excluding patients with underlying cancer of expected lifespan < 6 months are presented in Additional file 1: Table S4. On multivariable analyses, a higher NLR was consistently associated with higher in-hospital complications (OR 1.03; 95% CI 1.01–1.06, p = 0.015) and overall mortality (HR 1.02; 95% CI 1.00–1.02, p = 0.023). Subgroup analyses of overall mortality stratified by NLR level (NLR ≤ 5, 5 < NLR ≤ 12, NLR > 12) according to age, sex, and baseline LVEF are summarized in Additional file 1: Table S5. Overall, the estimates of adjusted HR increased in accordance with the increment of NLR value across the subgroups. No interaction was observed among age and sex subgroups, whereas a significant interaction was found between LVEF ≤ 40 and LVEF > 40% group (p-for-interaction = 0.018); those with initial LVEF > 40% presented a more prominent increase in adjusted HRs according to the increase of NLR value.

## Discussion

From our retrospective cohort of patients with TTS precede by physical triggers, our principal findings were as follows: (1) high NLR, high peak CRP, and reduced LVEF were associated with high IHCs; (2) older age and high NLR were related to a higher overall mortality rate; (3) notably, high NLR was a clinical marker that commonly correlated with both short- and long-term prognosis in patients with TTS preceded by physical triggers; and (4) particularly, patients with NLR > 12 harbor greater risks of mechanical ventilation, in-hospital mortality as well as 3.7-fold higher rates of overall mortality compared to those with NLR ≤ 5.

Our study population characteristics have several comparable features with patients from previous TTS studies. For example, the mean age (late 60 s) and the mean LVEF (slightly lower than 40%) are consistent with previous study populations. However, in the present study, the proportion of male patients was higher than in the prior research (37% vs. less than 20%) [1, 6, 10, 18]. Also, a substantial percentage of patients had terminal cancer (16.9%) and 27.8% of all subjects showed atypical ballooning (elsewhere reported at approximately 20%) [1, 2, 19]. The differences, possibly due to the severity of the underlying illness, might originate from the inclusion of TTS with physical stress only, which is associated with higher rates of adverse clinical outcomes than cases with emotional triggers [6, 10]. Indeed, we reported higher rates of IHCs (46.8%), which were largely due to higher rates of mechanical ventilation (39.8%) and in-hospital death (11.7%), together with higher overall mortality (13.6% per patient-year), compared to mechanical ventilation under 30%, in-hospital death up to 5%, and overall mortality 3.5% per patient-year reported in previous studies [8, 11]. However, the rate of malignant arrhythmia (11.7%) is comparable with the prior research reporting an 11.4% prevalence of life-threatening arrhythmia [15]. Also, an analysis of the US-nationwide TTS hospitalization database has reported event rate of mechanical ventilation, use of intra-aortic balloon pump, cardiac arrest, cardiogenic shock, and mortality near up to 40% [20]. Given the different definitions of IHC adopted in each research, the higher rate of our study’s IHC is not exceptional and the possible selection bias could explain the excess rate.

As TTS is now associated with considerable risks of complications and mortality not dissimilar to acute coronary syndrome [21], various features have been associated with short- and long-term outcomes. A recent study from a German, Italian, and Spanish TTS registry identified male sex, history of neurologic disorder, right ventricular involvement, and LVEF as independent predictors of IHCs [1]. Moreover, a Japanese multicenter TTS registry study revealed that high WBC counts and brain natriuretic peptide levels were associated with an adverse disease course during hospitalization [22]. Another observational study suggests that an LVEF cutoff of 38% and an LV global longitudinal strain cutoff of − 10% have incremental prognostic value in poor long-term survival [19], and a meta-regression analysis of patients discharged alive after TTS revealed old age, physical stressors, and atypical ballooning are significantly related to an unfavorable prognosis [6]. In addition to these factors, lower systolic blood pressure, Q-wave in the electrocardiogram, and high troponin levels at admission have been associated with IHCs [23]. Consistent with the prior findings, we verified the utility of known factors predicting outcomes of TTS preceded by physical triggers; reduced LVEF and high CRP levels as an indicator of unfavorable hospitalization course and old age for poor survival. Notably, we observed that NLR not only acts as a prognostic marker of adverse outcomes during hospitalization but also shows a predictive power for long-term mortality. Specifically, this is in line with the result of a recent Italian multicenter prospective study stating that an NLR > 5 is an independent predictor of IHCs in cases of TTS [24]. Our cutoff value of NLR (> 12) was higher than that of a prior study, possibly due to the limited inclusion of patients with TTS associated with physical events, and we validated the additive value of NLR as a hematologic index to indicate long-term mortality, as well.

Although the exact mechanism and pathogenesis of TTS remain unclarified, recent studies highlight the inflammatory nature of this disorder. In an experimental rodent model of catecholamine-induced Takotsubo-like cardiac dysfunction, early infiltration of neutrophils into myocardial tissue followed by a predominant influx of M1 pro-inflammatory macrophages without a conventional switch to M2 macrophages was observed and similar changes in post-mortem Takotsubo human hearts were also reported [25]. In addition, analyses of cardiac magnetic resonance imaging and blood monocytes with serum cytokines of patients with TTS concluded that TTS is characterized by macrophage-mediated myocardial inflammation and systemic peripheral inflammatory responses [26, 27]. Moreover, prior studies have suggested that a low-grade, chronic, and non-resolving inflammatory state might persist after the acute presentation of TTS [25, 26]. Both myocardial and systemic levels of inflammation might be represented as reduced LVEF, higher CRP, or higher NLR and account for the poor prognosis of TTS.

A high WBC count is known to be associated with an increased risk of mortality in ACS patients, as well [28]. One subtype of leukocytes, neutrophil, is the first inflammatory cell observed in myocardial damage and may contribute to atherosclerotic plaque destabilization, thrombosis, and endothelial cell dysfunction [25]. Lymphocyte has a regulatory function in an inflammatory response and could be reduced by an increase in cortisol and catecholamine. The subtypes of leukocytes and their prognostic role in the risk of many diseases, such as coronary artery disease and cancer, have been extensively reported [29, 30]. As high NLR on admission has a significant association with increased risks of both in-hospital and long-term mortality and major adverse cardiovascular events (MACE) in AMI [30], we expanded the previous predictive role of NLR to the clinical course in patients with TTS preceded by physical triggers.

According to analyses from a national inpatient sample database of the United States, there is a marked increase in the annual number of hospitalizations for TTS and an accompanied growing cost of care is expected [18, 20]. Given that TTS shows stable mortality rates over time and treatment is largely supportive [31], early risk stratification of patients according to known predictors and intensive monitoring with careful follow-up is warranted, especially in patients with physical triggers.

### Limitations

Our findings should be interpreted with the following limitations. First, selection bias might have been introduced since patient data were collected at tertiary referral hospitals where more critically ill patients were admitted. Second, this is a retrospective study; thus, baseline laboratory values and examination results might have been taken at different intervals from the initial recognition of TTS, and various clinical decisions might have influenced individual clinical outcomes. Moreover, there may be a variation in assay technique for complete blood cell counts and their subtypes due to the nature of a post-hoc analysis and the lack of standardized protocol. Third, our results require prospective validation in further studies. Finally, the optimal cutoff value of NLR with higher negative and positive predictive values to predict the prognosis of TTS should be evaluated in larger cohorts.

## Conclusion

In conclusion, NLR at initial recognition of the disorder is associated with an increased risk of both IHCs (malignant arrhythmia, need for mechanical circulatory support or mechanical ventilation, and in-hospital death) and overall mortality in patients of TTS preceded by physical triggers. The presence and severity of inflammatory response might play an important role in the clinical course of TTS. Intensive monitoring with careful follow-up would be warranted in TTS patients with physical stressors presenting higher NLR.

## Supplementary Information

**Additional file 1.** Supplementary Figures and Tables.

## Data Availability

The datasets used and/or analysed during the current study are available from the corresponding author on reasonable request.
