# Supplementary material for: Neutrophil-to-lymphocyte ratio as a predictor of in-hospital complications and overall mortality in Takotsubo syndrome preceded by physical triggers
Source: BMC Cardiovasc Disord. 2023 Jan 27;23:51. doi: 10.1186/s12872-023-03078-1 (PMC9881304; doi:10.1186/s12872-023-03078-1)
Supplement: Supplementary file 1 — Additional file 1. Supplementary Figures and Tables. [file 12872_2023_3078_MOESM1_ESM.docx]

**Supplementary Figures and Tables**

**Supplementary Figure 1**. Description of physical triggers and the examples of each category.

**Supplementary Figure 2**. Receiver operating characteristic curves demonstrating an area under the curve for in-hospital complications.

**Supplementary Figure 3**. The number of patients with the presence of individual components of IHCs stratified according to the value of neutrophil to lymphocyte ratio as 12.

**Supplementary Figure 4**. Kaplan–Meier survival curves of the patients of Takotsubo syndrome with physical triggers separated on the basis of neutrophil to lymphocyte ratio (NLR) values: low, NLR ≤ 12; and high, NLR > 12.

**Supplementary Table 1**. Baseline characteristics of study population according to the presence of in-hospital complications.

**Supplementary Table 2.** The general clinical features of takotsubo syndrome patients excluded for emotional triggers.

**Supplementary Table 3.** Follow-up echocardiographic evaluation.

**Supplementary Table 4.** Sensitivity analyses excluding patients with underlying cancer of expected lifespan <6 months.

**Supplementary Table 5**. Subgroup analyses of overall mortality according to age, sex, and left ventricular ejection fraction.

**
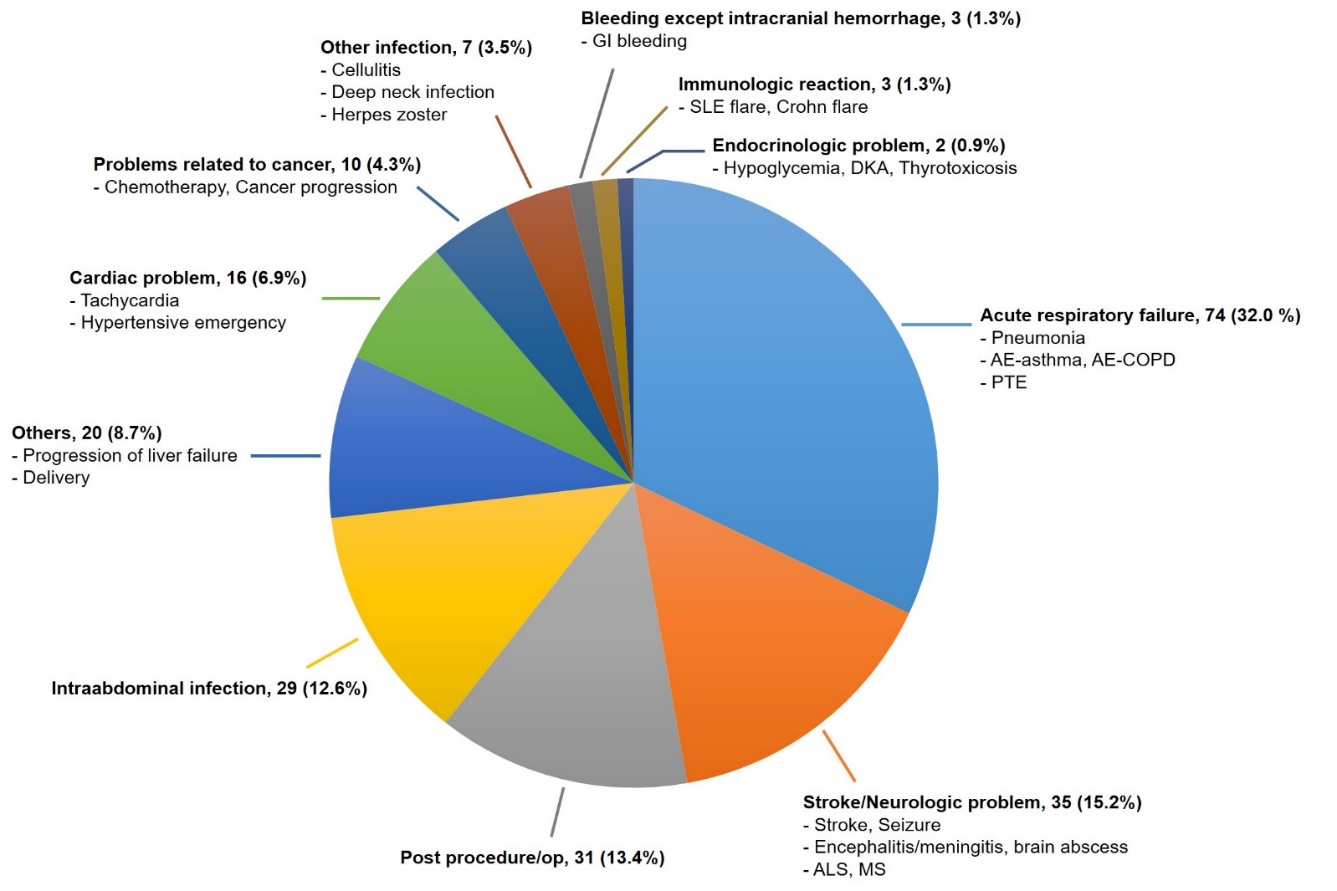
Supplementary Figure 1**. Description of physical triggers and the examples of each category.

Abbreviation: AE-asthma, acute exacerbation of asthma; AE-COPD, acute exacerbation of chronic obstructive pulmonary disease; PTE, pulmonary thromboembolism; ALS, amyotrophic lateral sclerosis; MS, multiple sclerosis; op, operation; GI, gastrointestinal; SLE, systemic lupus erythematosus; DKA, diabetic ketoacidosis.

**Supplementary Figure 2**. Receiver operating characteristic curves demonstrating an area under the curve for in-hospital complications.


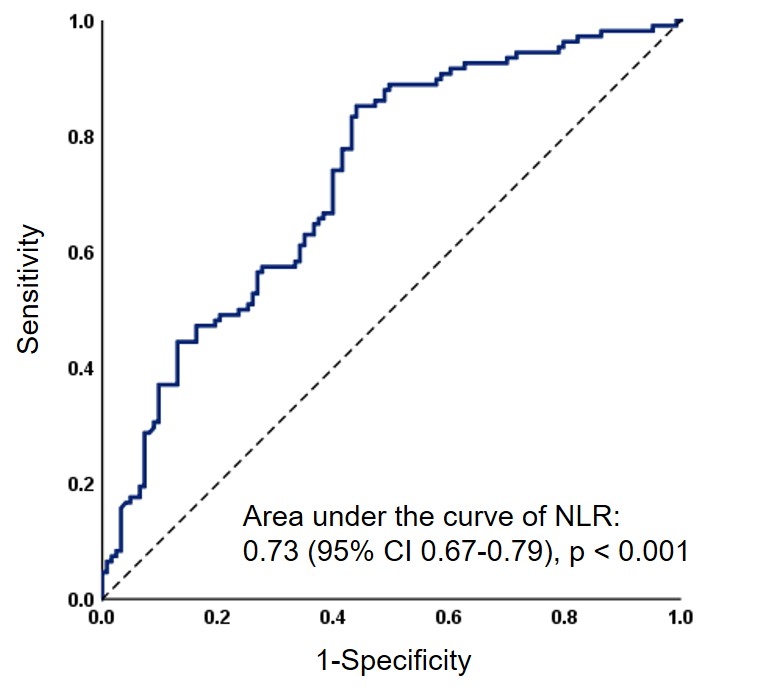


Abbreviation: NLR, neutrophil to lymphocyte rate; CI, confidence interval.

**Supplementary Figure 3**. The number of patients with the presence of individual components of IHCs stratified according to the value of neutrophil to lymphocyte ratio as 12.


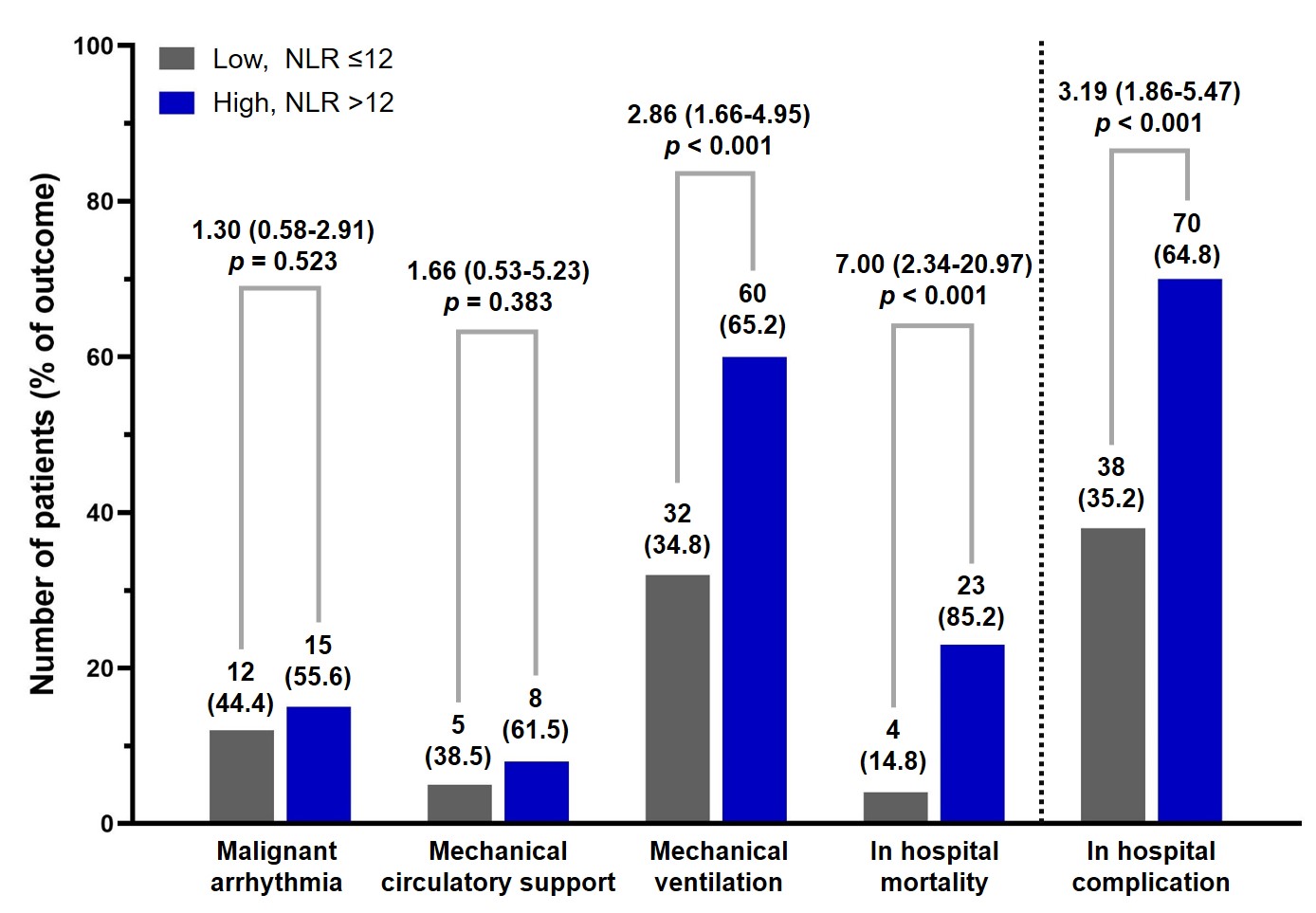


Abbreviation: NLR, neutrophil to lymphocyte ratio; CI, confidence interval.

**Supplementary Figure 4**. Kaplan–Meier survival curves of the patients of Takotsubo syndrome with physical triggers separated on the basis of neutrophil to lymphocyte ratio (NLR) values: low, NLR ≤ 12; and high, NLR > 12.


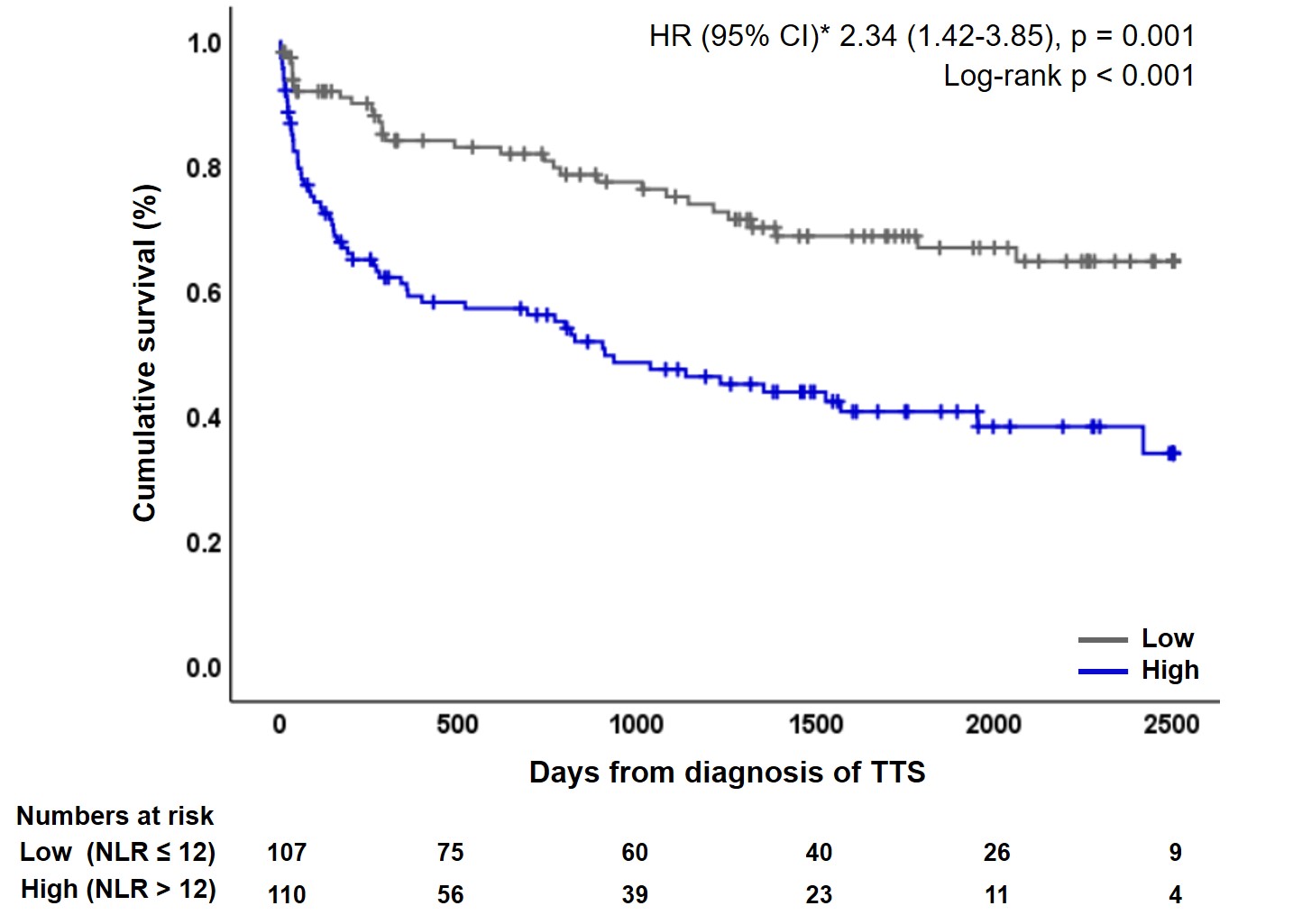


Abbreviation: TTS, takotsubo syndrome; NLR, neutrophil to lymphocyte ratio; HR, hazard ratio; CI, confidence interval; TnI, troponin I; LVEF, left ventricular ejection fraction.

* Adjusted for age, sex, cancer expected lifespan < 6 months, chest pain, peak TnI, peak CRP, LVEF, and atypical ballooning.

**Supplementary Table 1**. Baseline characteristics of study population according to the presence of in-hospital complications.

|  | Total | No In-hospital complication | In-hospital complication | p |
| --- | --- | --- | --- | --- |
| **No. of patients (%)** | 231 | 123 (53.2) | 108 (46.8) |  |
| **Demographics** |  |  |  |  |
| Age | 69.3 ± 11.6 | 69.7 ± 12.3 | 68.8 ± 10.8 | 0.547 |
| Sex |  |  |  |  |
| Male | 85 (36.8) | 33 (26.8) | 52 (48.1) | 0.001 |
| Female | 146 (63.2) | 90 (73.2) | 56 (51.9) |  |
| Height (cm) | 158.1 ± 8.9 (n=224) | 157.1 ± 8.8 (n=117) | 159.3 ± 8.9 (n=107) | 0.066 |
| Weight (kg) | 54.2 ± 11.1 (n=230) | 53.6 ± 11.2 (n=122) | 54.8 ± 10.9 (n=108) | 0.409 |
| Body mass index (kg/m2) | 21.6 ± 3.9 (n=224) | 21.7 ± 3.8 (n=117) | 21.5 ± 3.9 (n=107) | 0.770 |
| **Cardiovascular risk factors** |  |  |  |  |
| Hypertension | 100 (43.3) | 58 (47.2) | 42 (38.9) | 0.206 |
| Diabetes mellitus | 64 (27.7) | 37 (30.1) | 27 (25.0) | 0.389 |
| Dyslipidemia | 19 (8.2) | 11 (8.9) | 8 (7.4) | 0.672 |
| Current smoking | 25 (10.8) | 15 (12.2) | 10 (9.3) | 0.474 |
| **Comorbidities** |  |  |  |  |
| Coronary artery disease | 40 (17.3) | 23 (18.7) | 17 (15.7) | 0.553 |
| COPD or asthma | 22 (9.5) | 12 (9.8) | 10 (9.3) | 0.898 |
| ESRD | 19 (8.2) | 10 (8.1) | 9 (8.3) | 0.955 |
| Chronic liver disease | 10 (4.3) | 3 (2.4) | 7 (6.5) | 0.195 |
| Cancer expected lifespan < 6 months | 39 (16.9) | 15 (12.2) | 24 (22.2) | 0.042 |
| Hypo/Hyperthyroidism | 20 (8.7) | 12 (9.8) | 8 (7.4) | 0.527 |
| Neurologic disorders | 68 (29.4) | 37 (30.1) | 31 (28.7) | 0.819 |
| Cerebrovascular accident | 38 (16.5) | 24 (19.5) | 14 (13.0) | 0.180 |
| Seizure | 9 (3.9) | 7 (5.7) | 2 (1.9) | 0.180 |
| Neurodegenerative disease | 4 (1.7) | 1 (0.8) | 3 (2.8) | 0.342 |
| Others | 21 (9.1) | 9 (7.3) | 12 (11.1) | 0.317 |
| Psychiatric disorders | 10 (4.3) | 7 (5.7) | 3 (2.8) | 0.344 |
| **Trigger** |  |  |  |  |
| After procedure or operation | 31 (13.4) | 17 (13.8) | 14 (13.0) | 0.248 |
| Neurologic problems | 35 (15.2) | 23 (18.7) | 12 (11.1) |  |
| Others | 165 (71.4) | 83 (67.5) | 82 (75.9) |  |
| **Clinical presentation** |  |  |  |  |
| Chest pain | 62 (26.8) | 43 (35.0) | 19 (17.6) | 0.003 |
| Dyspnea | 84 (36.4) | 31 (25.2) | 53 (49.1) | < 0.001 |
| Shock | 47 (20.3) | 13 (10.6) | 34 (31.5) | < 0.001 |
| Others | 76 (32.9) | 48 (39.0) | 28 (25.9) | 0.035 |
| **Initial vital signs** |  |  |  |  |
| SBP (mmHg) | 110.4 ± 31.1  (n=231) | 117.4 ± 30.7  (n=123) | 102.3 ± 29.5  (n=108) | < 0.001 |
| DBP (mmHg) | 67.8 ± 17.5  (n=231) | 71.3 ± 17.4  (n=123) | 63.9 ± 16.7  (n=108) | 0.001 |
| MAP (mmHg) | 82.0 ± 20.9  (n=231) | 86.7 ± 20.7  (n=123) | 76.7 ± 19.9  (n=108) | < 0.001 |
| Heart rate (/min) | 98.1 ± 24.6  (n=231) | 92.5 ± 22.1  (n=123) | 104.5 ± 25.8  (n=108) | < 0.001 |
| **Cardiac biomarkers** |  |  |  |  |
| Initial CK (IU/L) | 103.5 (57.8-220.3)  (n=218) | 99.0 (57.5-162.5)  (n=113) | 115.0 (58.5-244.5)  (n=105) | 0.218 |
| Initial CKMB (ng/mL) | 4.2 (1.8-11.8)  (n=225) | 3.4 (1.8-9.7)  (n=117) | 5.5 (1.9-14.2)  (n=108) | 0.089 |
| Initial Troponin I (ng/mL) | 0.4 (0.1-2.2)  (n=225) | 0.4 (0.1-2.2)  (n=117) | 0.5 (0.1-2.3)  (n=108) | 0.324 |
| Peak CK (IU/L) | 162.0 (74.0-404.0)  (n=218) | 143.5 (68.5-309.5)  (n=113) | 213.0 (83.0-595.0)  (n=105) | 0.007 |
| Peak CKMB (ng/mL) | 9.3 (3.9-19.6)  (n=225) | 6.6 (2.9-15.0)  (n=117) | 14.2 (5.6-26.4)  (n=108) | < 0.001 |
| Peak Troponin I (ng/mL) | 2.0 (0.4-5.4)  (n=225) | 1.3 (0.3-3.4)  (n=117) | 2.9 (1.0-8.5)  (n=108) | < 0.001 |
| Initial BNP (pg/mL) | 657.0 (194.5-1775.0)  (n=81) | 511.0 (141.0-1514.8)  (n=40) | 749.0 (281.0-2241.0)  (n=41) | 0.132 |
| Peak BNP (pg/mL) | 696.0 (212.0-2400.0)  (n=81) | 511.0 (166.8-1566.5)  (n=40) | 921.0 (369.5-3036.0)  (n=41) | 0.076 |
| **Inflammatory markers** |  |  |  |  |
| Initial CRP (mg/dL) | 4.3 (0.7-14.1)  (n=227) | 3.1 (0.5-7.0)  (n=119) | 6.7 (1.6-19.4)  (n=108) | 0.001 |
| Peak CRP (mg/dL) | 10.5 (3.9-19.9)  (n=227) | 5.9 (1.3-13.5)  (n=119) | 17.9 (8.0-26.0)  (n=108) | <0.001 |
| Initial WBC (/μL) | 9850.0 (6900.0-14620.0)  (n=231) | 8800.0 (6680.0-11220.0)  (n=123) | 12645.0 (7625.0-18430.0)  (n=108) | <0.001 |
| Peak WBC (/μL) | 15020.0 (9460.0-21610.0)  (n=231) | 10610.0 (8250.0-16500.0)  (n=123) | 20775.0 (14565.0-27340.0)  (n=108) | <0.001 |
| NLR | 12.0 (5.4-22.5)  (n=231) | 6.7 (4.0-16.3)  (n=123) | 16.4 (9.6-32.1)  (n=108) | <0.001 |
| PLR | 178.8 (98.2-303.3)  (n=148) | 164.5 (91.2-299.4)  (n=79) | 186.5 (105.9-322.1)  (n=69) | 0.096 |
| **Other laboratory values** |  |  |  |  |
| D-dimer (μg/mL) | 2.7 (1.3-5.8)  (n=133) | 1.7 (0.8-3.6)  (n=63) | 4.4 (1.8-7.8)  (n=70) | <0.001 |
| BUN (mg/dL) | 18.0 (12.0-31.0)  (n=230) | 16.0 (11.0-26.0)  (n=122) | 22.5 (15.0-38.0)  (n=108) | <0.001 |
| Cr (mg/dL) | 0.9 (0.7-1.5)  (n=230) | 0.8 (0.6-1.1)  (n=122) | 1.0 (0.7-1.6)  (n=108) | 0.020 |
| TG (mg/dL) | 86.5 (61.0-130.0)  (n=90) | 90.0 (61.0-133.0)  (n=59) | 80.0 (57.8-113.8)  (n=31) | 0.420 |
| Chol (mg/dL) | 131.0 (104.0-174.0)  (n=224) | 149.0 (124.0-189.0)  (n=118) | 131.0 (97.8-151.3)  (n=106) | <0.001 |
| HDL-C (mg/dL) | 40.0 (28.0-55.0)  (n=89) | 42.0 (29.0-61.0)  (n=59) | 37.0 (26.5-47.0)  (n=30) | 0.167 |
| LDL-C (mg/dL) | 78.0 (60.0-109.0)  (n=90) | 90.0 (62.0-112.0)  (n=60) | 70.0 (45.8-87.4)  (n=30) | 0.005 |
| **ECG findings** |  |  |  |  |
| **rhythm** |  |  |  |  |
| Sinus rhythm | 195 (84.4) | 101 (82.1) | 94 (87.0) | 0.585 |
| Atrial fibrillation | 21 (9.1) | 13 (10.6) | 8 (7.4) |  |
| Others | 15 (6.5) | 9 (7.3) | 6 (5.6) |  |
| Details |  |  |  |  |
| AV block | 3 (1.3) | 1 (0.8) | 2 (1.9) | 0.602 |
| ST-segment elevation | 59 (25.5) | 21 (17.1) | 38 (35.2) | 0.002 |
| ST-segment depression | 16 (6.9) | 8 (6.5) | 8 (7.4) | 0.787 |
| T-wave inversion | 97 (42.0) | 58 (47.2) | 39 (36.1) | 0.090 |
| Left bundle branch block | 7 (3.0) | 2 (1.6) | 5 (4.6) | 0.256 |
| QTc prolongation | 169 (73.2) | 89 (72.4) | 80 (74.1) | 0.769 |
| QTc (ms) | 484.0 ± 61.8  (n=231) | 479.6 ± 62.5 (n=123) | 488.9 ± 60.8 (n=108) | 0.255 |
| **Echocardiographic findings** |  |  |  |  |
| LVEF (%) | 39.7 ± 11.0  (n=231) | 42.0 ± 9.9  (n=123) | 37.0 ± 11.6 (n=108) | 0.001 |
| LVEF ≤ 40% | 116 (50.2) | 51 (41.5) | 65 (60.2) | 0.005 |
| LVEF > 40% | 115 (49.8) | 72 (58.5) | 43 (39.8) |  |
| Regional wall motion abnormality |  |  |  |  |
| Apical ballooning | 169 (73.2) | 89 (72.4) | 80 (74.1) | 0.274 |
| Midventricular | 19 (8.2) | 11 (8.9) | 8 (7.4) |  |
| Basal or inverted (Reverse) | 12 (5.2) | 4 (3.3) | 8 (7.4) |  |
| Biventricular | 12 (5.2) | 5 (4.1) | 7 (6.5) |  |
| Focal | 18 (7.8) | 13 (10.6) | 5 (4.6) |  |
| **Clinical outcomes** |  |  |  |  |
| Malignant arrhythmia | 27 (11.7) | N/A | 27 (25.0) |  |
| Mechanical circulatory support | 13 (5.6) | N/A | 13 (12.0) |  |
| Mechanical ventilation | 92 (39.8) | N/A | 92 (85.2) |  |
| In hospital mortality | 27 (11.7) | N/A | 27 (25.0) |  |

Abbreviation: COPD, chronic obstructive pulmonary disease; ESRD, end stage renal disease; SBP, systolic blood pressure; DBP, diastolic blood pressure; MAP, mean arterial pressure; CK, creatine kinase; CKMB, Creatine kinase-MB; BNP, B type natriuretic peptide; CRP, C-reactive protein; WBC, white blood cell; NLR, neutrophil to lymphocyte ratio; PLR, platelet to lymphocyte ratio; BUN, blood urea nitrogen; Cr, creatinine; TG, triglyceride; Chol, cholesterol; HDL-C, high density lipoprotein cholesterol; LDL-C, low density lipoprotein cholesterol; LVEF, left ventricular ejection fraction.

**Supplementary Table 2.** The general clinical features of Takotsubo syndrome patients excluded for emotional triggers.

|  | Emotional triggers | Physical triggers | P-value |
| --- | --- | --- | --- |
|  | (n=23) | (n=231) |  |
| **Age** | 65.5 ± 13.5 | 69.3 ± 11.6 | 0.140 |
| **Male** | 5 (21.7%) | 85 (36.8%) | 0.150 |
| **Body mass index (kg/m2)** | 23.9 ± 3.4 | 21.6 ± 3.9 | 0.019 |
| **Comorbidities** |  |  |  |
| Hypertension | 16 (69.6%) | 100 (43.3%) | 0.016 |
| Diabetes mellitus | 5 (21.7%) | 64 (27.7%) | 0.540 |
| Dyslipidemia | 5 (21.7%) | 19 (8.2%) | 0.035 |
| Coronary artery disease | 0 (0.0%) | 40 (17.3%) | 0.030 |
| COPD or asthma | 1 (5.6%) | 22 (9.5%) | 0.580 |
| ESRD | 1 (4.3%) | 19 (8.2%) | 0.510 |
| Chronic liver disease | 2 (11.1%) | 10 (4.3%) | 0.200 |
| Cancer expected lifespan < 6 months | 1 (4.3%) | 39 (16.9%) | 0.120 |
| Neurologic disorders | 3 (13.0%) | 68 (29.4%) | 0.095 |
| Psychiatric disorders | 2 (11.1%) | 10 (4.3%) | 0.200 |
| **Hematologic index** |  |  |  |
| NLR | 3.9 (1.9-5.8) | 12.0 (5.4-22.5) | 0.008 |
| PLR | 178.8 (98.2-303.3) | 130.7 (88.5-203.9) | 0.140 |
| **LVEF (%)** | 45.7 ± 14.1 | 39.7 ± 11.0 | 0.016 |
| **ICU admission** | 5 (27.8%) | 120 (51.9%) | 0.048 |
| APACHE_II | 10.8 ± 4.5 | 20.3 ± 8.2 | 0.013 |
| SOFA | 3.8 ± 2.5 | 7.6 ± 3.6 | 0.026 |
| SAPS II | 25.6 ± 10.7 | 46.6 ± 15.0 | 0.003 |
| **In hospital mortality** | 0 (0.0%) | 27 (11.7%) | 0.083 |

Abbreviation: COPD, chronic obstructive pulmonary disease; ESRD, end-stage renal disease; LVEF, left ventricular ejection fraction; ICU, intensive care unit; APACHE II, acute physiology and chronic health evaluation **II;** SOFA, sequential organ failure assessment; SAPS II, simplified acute physiology score II.

**Supplementary Table 3.** Follow-up echocardiographic evaluation.

|  | Total | In-hospital mortality | | p-value |
| --- | --- | --- | --- | --- |
|  | N=167 | No, N=154 | Yes, N=13 |  |
| Baseline LVEF (%) | 39.3 ± 10.5 | 39.8 ± 10.4 | 33.0 ± 10.1 | 0.025 |
| Follow up interval (days) | 20.5 (8.4-85.6) | 21.4 (9.0-100.5) | 9.0 (6.6-20.4) | 0.11 |
| Follow up LVEF (%) | 54.6 ± 10.0 | 55.1 ± 10.0 | 48.3 ± 9.2 | 0.019 |
| Improvement in RWMA | 149 (89.2%) | 138 (89.6%) | 11 (84.6%) | 0.58 |
| Improvement in LVEF | 155 (92.8%) | 144 (93.5%) | 11 (84.6%) | 0.23 |
| Delta (Δ) of LVEF (%) | 15.3 ± 11.9 | 15.3 ± 11.7 | 15.3 ± 14.7 | 0.99 |

Abbreviation: NLR, neutrophil to lymphocyte ratio; HR, hazard ratio; CI, confidence interval; LVEF, left ventricular ejection fraction.

**Supplementary Table 4.** Sensitivity analyses excluding patients with underlying cancer of expected lifespan <6 months.

| Multivariable analyses | In-hospital complications | | Overall mortality | |
| --- | --- | --- | --- | --- |
|  | β (95% CI) | P Value | Hazard Ratio (95% CI) | P Value |
| Age* | 0.93 (0.68-1.25) | 0.610 | 1.36 (1.04-1.78) | 0.024 |
| Male sex | 1.53 (0.74-3.15) | 0.250 | 2.00 (1.17-3.41) | 0.011 |
| Chest pain | 0.34 (0.15-0.77) | 0.010 | 0.97 (0.55-1.72) | 0.916 |
| Peak troponin I | 1.03 (0.99-1.06) | 0.103 | 1.00 (0.99-1.00) | 0.479 |
| NLR | 1.03 (1.01-1.06) | 0.015 | 1.02 (1.00-1.02) | 0.023 |
| Peak CRP | 1.05 (1.01-1.10) | 0.014 | 0.99 (0.96-1.03) | 0.705 |
| LVEF | 0.97 (0.94-1.00) | 0.080 | 0.99 (0.96-1.02) | 0.426 |
| Atypical ballooning | 1.04 (0.47-2.27) | 0.928 | 1.03 (0.60-1.78) | 0.912 |

Abbreviation: CI, confidential interval; NLR, neutrophil to lymphocyte ratio; CRP, C-reactive protein; LVEF, left ventricular ejection fraction.

* for a 10-year increase.

**Supplementary Table 5**. Subgroup analyses of overall mortality according to age, sex, and left ventricular ejection fraction.

| **Subgroup** | **NLR group** | **n** | **HR* (95% CI)** | **p** | **p-for-interaction** |
| --- | --- | --- | --- | --- | --- |
| Age<70 years | NLR≤5 | 25 | 1 (Reference) |  | 0.496 |
|  | 5<NLR≤12 | 26 | 2.223 (0.554-8.918) | 0.26 |  |
|  | NLR>12 | 59 | 5.501 (1.547-19.562) | 0.008 |  |
| Age≥70 years | NLR≤5 | 28 | 1 (Reference) |  |  |
|  | 5<NLR≤12 | 37 | 2.406 (0.836-6.925) | 0.104 |  |
|  | NLR>12 | 56 | 4.017 (1.448-11.146) | 0.008 |  |
| Male | NLR≤5 | 11 | 1 (Reference) |  | 0.124 |
|  | 5<NLR≤12 | 23 | 1.306 (0.321-5.326) | 0.709 |  |
|  | NLR>12 | 51 | 1.339 (0.336-5.327 ) | 0.679 |  |
| Female | NLR≤5 | 42 | 1 (Reference) |  |  |
|  | 5<NLR≤12 | 40 | 1.888 (0.662-5.389) | 0.235 |  |
|  | NLR>12 | 64 | 6.087(2.358-15.709) | <0.001 |  |
| LVEF≤40% | NLR≤5 | 23 | 1 (Reference) |  | 0.018 |
|  | 5<NLR≤12 | 32 | 0.979 (0.323-2.968) | 0.969 |  |
|  | NLR>12 | 61 | 1.983 (0.697-5.636 ) | 0.199 |  |
| LVEF>40% | NLR≤5 | 30 | 1 (Reference) |  |  |
|  | 5<NLR≤12 | 31 | 5.045 (1.365-18.644) | 0.015 |  |
|  | NLR>12 | 54 | 8.529 (2.456-29.619 ) | 0.001 |  |

* Adjusted for age, sex, cancer expected lifespan < 6 months, chest pain, peak TnI, peak CRP, LVEF, and atypical ballooning.

Abbreviation: NLR, neutrophil to lymphocyte ratio; HR, hazard ratio; CI, confidence interval; LVEF, left ventricular ejection fraction.
